# Supplementary material for: Positive Effects of Exercise Intervention without Weight Loss and Dietary Changes in NAFLD-Related Clinical Parameters: A Systematic Review and Meta-Analysis
Source: Nutrients. 2021 Sep 8;13(9):3135. doi: 10.3390/nu13093135 (PMC8466505; doi:10.3390/nu13093135)
Supplement: Supplementary file 1 [file nutrients-13-03135-s001.zip › nutrients-1367107-supplementary.pdf]

Supplementary Materials:

Supplement S1: PRISMA check list:

Section and Topic	Item #	Checklist item	Location where item is reported
**TITLE**			
Title	1	Identify the report as a systematic review.	Title
**ABSTRACT**			
Abstract	2	See the PRISMA 2020 for Abstracts checklist.	Abstract
**INTRODUCTION**			
Rationale	3	Describe the rationale for the review in the context of existing knowledge.	Introduction
Objectives	4	Provide an explicit statement of the objective(s) or question(s) the review addresses.	Introduction
**METHODS**			
Eligibility criteria	5	Specify the inclusion and exclusion criteria for the review and how studies were grouped for the syntheses.	Methods
Information sources	6	Specify all databases, registers, websites, organisations, reference lists and other sources searched or consulted to identify studies. Specify the date when each source was last searched or consulted.	Methods
Search strategy	7	Present the full search strategies for all databases, registers and websites, including any filters and limits used.	Methods
Selection process	8	Specify the methods used to decide whether a study met the inclusion criteria of the review, including how many reviewers screened each record and each report retrieved, whether they worked independently, and if applicable, details of automation tools used in the process.	Methods
Data collection process	9	Specify the methods used to collect data from reports, including how many reviewers collected data from each report, whether they worked independently, any processes for obtaining or confirming data from study investigators, and if applicable, details of automation tools used in the process.	Methods
Data items	10a	List and define all outcomes for which data were sought. Specify whether all results that were compatible with each outcome domain in each study were sought (e.g. for all measures, time points, analyses), and if not, the methods used to decide which results to collect.	Methods
	10b	List and define all other variables for which data were sought (e.g. participant and intervention characteristics, funding sources). Describe any assumptions made about any missing or unclear information.	Methods
Study risk of bias assessment	11	Specify the methods used to assess risk of bias in the included studies, including details of the tool(s) used, how many reviewers assessed each study and whether they worked independently, and if applicable, details of automation tools used in the process.	Methods
Effect measures	12	Specify for each outcome the effect measure(s) (e.g. risk ratio, mean difference) used in the synthesis or presentation of results.	Methods
Synthesis methods	13a	Describe the processes used to decide which studies were eligible for each synthesis (e.g. tabulating the study intervention characteristics and comparing against the planned groups for each synthesis (item #5)).	Methods
	13b	Describe any methods required to prepare the data for presentation or synthesis, such as handling of missing summary statistics, or data conversions.	Methods
	13c	Describe any methods used to tabulate or visually display results of individual studies and syntheses.	Methods
	13d	Describe any methods used to synthesize results and provide a rationale for the choice(s). If meta-analysis was performed, describe the model(s), method(s) to identify the presence and extent of statistical heterogeneity, and software package(s) used.	Methods
	13e	Describe any methods used to explore possible causes of heterogeneity among study results (e.g. subgroup analysis, meta-regression).	Methods
	13f	Describe any sensitivity analyses conducted to assess robustness of the synthesized results.	Methods

Reporting bias assessment	14	Describe any methods used to assess risk of bias due to missing results in a synthesis (arising from reporting biases).	Methods
Certainty assessment	15	Describe any methods used to assess certainty (or confidence) in the body of evidence for an outcome.	Methods
**RESULTS**			
Study selection	16a	Describe the results of the search and selection process, from the number of records identified in the search to the number of studies included in the review, ideally using a flow diagram.	Results
	16b	Cite studies that might appear to meet the inclusion criteria, but which were excluded, and explain why they were excluded.	Results
Study characteristics	17	Cite each included study and present its characteristics.	Results
Risk of bias in studies	18	Present assessments of risk of bias for each included study.	Results
Results of individual studies	19	For all outcomes, present, for each study: (a) summary statistics for each group (where appropriate) and (b) an effect estimate and its precision (e.g. confidence/credible interval), ideally using structured tables or plots.	Results
Results of syntheses	20a	For each synthesis, briefly summarise the characteristics and risk of bias among contributing studies.	Results
	20b	Present results of all statistical syntheses conducted. If meta-analysis was done, present for each the summary estimate and its precision (e.g. confidence/credible interval) and measures of statistical heterogeneity. If comparing groups, describe the direction of the effect.	Results
	20c	Present results of all investigations of possible causes of heterogeneity among study results.	Results
	20d	Present results of all sensitivity analyses conducted to assess the robustness of the synthesized results.	Results
Reporting biases	21	Present assessments of risk of bias due to missing results (arising from reporting biases) for each synthesis assessed.	Results
Certainty of evidence	22	Present assessments of certainty (or confidence) in the body of evidence for each outcome assessed.	Results
**DISCUSSION**			
Discussion	23a	Provide a general interpretation of the results in the context of other evidence.	Discussion
	23b	Discuss any limitations of the evidence included in the review.	Discussion
	23c	Discuss any limitations of the review processes used.	Discussion
	23d	Discuss implications of the results for practice, policy, and future research.	Discussion
**OTHER INFORMATION**			
Registration and protocol	24a	Provide registration information for the review, including register name and registration number, or state that the review was not registered.	Methods, Abstract
	24b	Indicate where the review protocol can be accessed, or state that a protocol was not prepared.	Methods
	24c	Describe and explain any amendments to information provided at registration or in the protocol.	Methods
Support	25	Describe sources of financial or non-financial support for the review, and the role of the funders or sponsors in the review.	After conclusion under funding
Competing interests	26	Declare any competing interests of review authors.	Declaration
Availability of data, code and other materials	27	Report which of the following are publicly available and where they can be found: template data collection forms; data extracted from included studies; data used for all analyses; analytic code; any other materials used in the review.	Data Availability Statement:

<u>Supplement S2:</u> Search terms

Search date: February 13, 2020

PubMed Advanced search

#1 "non-alcoholic fatty liver disease"[mh] OR "non alcoholic fatty liver"[tw] OR "nonalcoholic fatty liver"[tw] OR nafld[tw] OR nash[tw] OR steatohepatitis[tw] OR steatitis[tw] OR steatosis[tw] OR "liver cirrhosis"[mh] OR "liver cirrhosis"[tw] OR "hepatic cirrhosis"[tw] OR "liver fibrosis"[tw] OR "hepatic fibrosis"[tw]

139,933

#2 exercise[mh] OR sports[mh] OR "physical activity"[tw] OR "physical activities"[tw] OR exercis*[tw] OR sport*[tw] OR gym[tw] OR gyms[tw] OR gymnastic*[tw] OR aerobic*[tw] OR training[tw] OR hiit[tw] OR walk*[tw] OR jogging[tw] OR jogger*[tw] OR running[tw] OR runner*[tw] OR marathon[tw] OR swim*[tw] OR climb*[tw] OR athletic*[tw] OR bicycl*[tw] OR football[tw] OR soccer[tw] OR baseball[tw] OR basketball[tw] OR volleyball[tw] OR skiing[tw] OR skating[tw] OR hockey[tw] OR "weight lifting"[tw] OR weightlift*[tw]

1,152,446

#3 #1 AND #2

2,582

#4 "gastrointestinal microbiome"[mh] OR "gastrointestinal microbiome"[tw] OR "enteric bacteria"[tw] OR "gastric microbiome"[tw] OR "gastrointestinal flora"[tw] OR "gastrointestinal microbial community"[tw] OR "gastrointestinal microbiota"[tw] OR "gastrointestinal microflora"[tw] OR "gut flora"[tw] OR "gut microbiome"[tw] OR "gut microbiota"[tw] OR "gut microflora"[tw] OR "intestinal flora"[tw] OR "intestinal microbiome"[tw] OR "intestinal microbiota"[tw] OR "intestinal microflora"[tw]

40,530

#5 "gastrointestinal tract/metabolism"[mh] OR "gastrointestinal metabolite"[tw] OR "gastrointestinal metabolites"[tw] OR "intestinal metabolite"[tw] OR "intestinal metabolites"[tw] OR "gut metabolite"[tw] OR "gut metabolites"[tw] OR "gastric metabolite"[tw] OR "gastric metabolites"[tw] OR "gastrointestinal metabolism"[tw] OR "intestinal metabolism"[tw] OR "gut metabolism"[tw] OR "gastric metabolism"[tw]

129,741

#6 "liver fat"[tw] OR "liver fats"[tw] OR "liver lipid"[tw] OR "liver lipids"[tw] OR "liver stiffness"[tw] OR "hepatic fat"[tw] OR "hepatic fats"[tw] OR "hepatic lipid"[tw] OR "hepatic lipids"[tw] OR "hepatic stiffness"[tw] OR "liver adiposity"[tw] OR "hepatic adiposity"[tw]

13,648

#7 "liver/enzymology"[mh] OR "liver enzyme"[tw] OR "liver enzymes"[tw] OR "hepatic enzyme"[tw] OR "hepatic enzymes"[tw] OR alat[tw] OR asat[tw] OR ggt[tw]

101,819

#8 "lipid metabolism"[mh] OR "lipid metabolism"[tw] OR "glucose/metabolism"[mh] OR "blood glucose/metabolism"[mh] OR "glucose metabolism"[tw] OR "fasting glucose"[tw] OR "homa ir"[tw] OR hba1c[tw]

324,844

#9 "body weight"[mh] OR "body composition"[mh] OR "waist circumference"[mh] OR "body mass index"[mh] OR "body weight"[tw] OR "body composition"[tw] OR "waist circumference"[tw] OR "body mass index"[tw] OR bmi[tw] OR "fat mass"[tw]

783,008

#10 "cardiorespiratory fitness"[mh] OR "physical fitness"[mh] OR "exercise test"[mh] OR "fitness test"[tw] OR "fitness tests"[tw] OR "fitness testing"[tw] OR "exercise test"[tw] OR "exercise tests"[tw] OR "exercise testing"[tw] OR "respiratory test"[tw] OR "respiratory tests"[tw] OR "respiratory testing"[tw]

97,748

#11 #4 OR #5 OR #6 OR #7 OR #8 OR #9 OR #10

1,361,882

#12 #3 AND #11

1,338

#13 "randomized controlled trial"[pt] OR "randomized controlled trials as topic"[mh] OR "randomized controlled"[tw] OR "randomised controlled"[tw] OR "randomized control"[tw] OR "randomised control"[tw] OR "random control"[tw] OR "randomized trial"[tw] OR "randomised trial"[tw] OR "controlled trial"[tw] OR "randomized trials"[tw] OR "randomised trials"[tw] OR "controlled trials"[tw] OR "randomized study"[tw] OR "randomized studies"[tw] OR "randomised study"[tw] OR "randomised studies"[tw] OR "controlled study"[tw] OR "controlled studies"[tw] OR rct[tw] OR rcts[tw] OR random*[tw]
1,380,623
#14 #12 AND #13
273
#15 #14 AND english[la]
266

Scopus Advanced search
#1 TITLE-ABS-KEY("non alcoholic fatty liver*" OR "nonalcoholic fatty liver*" OR nafld OR nash OR steatohepatitis OR steatitis OR steatosis OR "liver cirrhosis" OR "hepatic cirrhosis" OR "liver fibrosis" OR "hepatic fibrosis")
229,315
#2 TITLE-ABS-KEY("physical activit*" OR exercis* OR sport* OR gym* OR aerobic* OR training OR hiit OR walk* OR jog* OR run* OR marathon OR swim* OR climb* OR athletic* OR bicycl* OR football OR soccer OR baseball OR basketball OR volleyball OR skiing OR skating OR hockey OR "weight lifting" OR weightlift*)
3,432,245
#3 #1 AND #2
8,273
#4 TITLE-ABS-KEY("gastrointestinal microbiome" OR "enteric bacteria" OR "gastric microbiome" OR "gastrointestinal flora" OR "gastrointestinal microbial community" OR "gastrointestinal microbiota" OR "gastrointestinal microflora" OR "gut flora" OR "gut microbiome" OR "gut microbiota" OR "gut microflora" OR "intestinal flora" OR "intestinal microbiome" OR "intestinal microbiota" OR "intestinal microflora")
48,039
#5 TITLE-ABS-KEY("gastrointestinal tract metaboli*" OR "gastrointestinal metaboli*" OR "intestinal metaboli*" OR "gut metaboli*" OR "gastric metaboli*")
1,559
#6 TITLE-ABS-KEY("liver fat*" OR "liver lipid*" OR "liver stiffness" OR "hepatic fat*" OR "hepatic lipid*" OR "hepatic stiffness" OR "liver adiposity" OR "hepatic adiposity")
20,686
#7 TITLE-ABS-KEY("liver enzym*" OR "hepatic enzym*" OR alat OR asat OR ggt)
53,168
#8 TITLE-ABS-KEY("lipid metabolism" OR "glucose metabolism" OR "fasting glucose" OR "homa ir" OR hba1c)
224,638
#8 TITLE-ABS-KEY("body weight" OR "body composition" OR "waist circumference" OR "body mass index" OR bmi OR "fat mass")
814,468
#10 TITLE-ABS-KEY("cardiorespiratory fitness" OR "physical fitness" OR "exercise test*" OR "fitness test*" OR "respirat* test*")
120,861
#11 #4 OR #5 OR #6 OR #7 OR #8 OR #9 OR #10
1,193,216
#12 #3 AND #11
2,118

#13 TITLE-ABS-KEY("random* control*" OR "random* trial*" OR "control* trial*" OR "random* stud*" OR "control* stud*" OR rct OR rcts)
6,612,829
#14 #12 AND #13
894
#15 #14 AND LANGUAGE(english)
871

Web of Science Advanced search
#1 TS=("non alcoholic fatty liver*" OR "nonalcoholic fatty liver*" OR nafld OR nash OR steatohepatitis OR steatitis OR steatosis OR "liver cirrhosis" OR "hepatic cirrhosis" OR "liver fibrosis" OR "hepatic fibrosis")
118,067
#2 TS=("physical activit*" OR exercis* OR sport* OR gym* OR aerobic* OR training OR hiit OR walk* OR jog* OR run* OR marathon OR swim* OR climb* OR athletic* OR bicycl* OR football OR soccer OR baseball OR basketball OR volleyball OR skiing OR skating OR hockey OR "weight lifting" OR weightlift*)
2,081,706
#3 #1 AND #2
5,025
#4 TS=("gastrointestinal microbiome" OR "enteric bacteria" OR "gastric microbiome" OR "gastrointestinal flora" OR "gastrointestinal microbial community" OR "gastrointestinal microbiota" OR "gastrointestinal microflora" OR "gut flora" OR "gut microbiome" OR "gut microbiota" OR "gut microflora" OR "intestinal flora" OR "intestinal microbiome" OR "intestinal microbiota" OR "intestinal microflora")
49,960
#5 TS=("gastrointestinal tract metaboli*" OR "gastrointestinal metaboli*" OR "intestinal metaboli*" OR "gut metaboli*" OR "gastric metaboli*")
1,369
#6 TS=("liver fat*" OR "liver lipid*" OR "liver stiffness" OR "hepatic fat*" OR "hepatic lipid*" OR "hepatic stiffness" OR "liver adiposity" OR "hepatic adiposity")
18,161
#7 TS=("liver enzym*" OR "hepatic enzym*" OR alat OR asat OR ggt)
24,373
#8 TS=("lipid metabolism" OR "glucose metabolism" OR "fasting glucose" OR "homa ir" OR hba1c)
132,598
#9 TS=(weight OR "body composition" OR "waist circumference" OR "body mass index" OR bmi OR "fat mass")
1,701,653
#10 TS=("cardiorespiratory fitness" OR "physical fitness" OR "exercise test*" OR "fitness test*" OR "respirat* test*")
42,908
#11 #4 OR #5 OR #6 OR #7 OR #8 OR #9 OR #10
1,901,891
#12 #3 AND #11
1,605
#13 TS=("random* control*" OR "random* trial*" OR "control* trial*" OR "random* stud*" OR "control* stud*" OR rct OR rcts)
752,330
#14 #12 AND #13
260
#15 #14 AND LANGUAGE: (English)
249

Cochrane Database of Systematic Reviews
Issue 2 of 12, February 2020
Cochrane Central Register of Controlled Trials
Issue 2 of 12, February 2020

#1 "non alcoholic fatty liver*" OR "nonalcoholic fatty liver*" OR nafld OR nash OR steatohepatitis OR steatitis OR steatosis OR "liver cirrhosis" OR "hepatic cirrhosis" OR "liver fibrosis" OR "hepatic fibrosis"
11,532
#2 "physical activit*" OR exercis* OR sport* OR gym* OR aerobic* OR training OR hiit OR walk* OR jog* OR run* OR marathon OR swim* OR climb* OR athletic* OR bicycl* OR football OR soccer OR baseball OR basketball OR volleyball OR skiing OR skating OR hockey OR "weight lifting" OR weightlift*
198,196
#3 1 AND #2
875
#4 "gastrointestinal microbiome" OR "enteric bacteria" OR "gastric microbiome" OR "gastrointestinal flora" OR "gastrointestinal microbial community" OR "gastrointestinal microbiota" OR "gastrointestinal microflora" OR "gut flora" OR "gut microbiome" OR "gut microbiota" OR "gut microflora" OR "intestinal flora" OR "intestinal microbiome" OR "intestinal microbiota" OR "intestinal microflora"
4015
#5 (gastrointestin* OR intestin* OR gastric OR gut) AND metaboli*
15,539
#6 "liver fat*" OR "liver lipid*" OR "liver stiffness" OR "hepatic fat*" OR "hepatic lipid*" OR "hepatic stiffness" OR "liver adiposity" OR "hepatic adiposity"
1,453
#7 "liver enzym*" OR "hepatic enzym*" OR alat OR asat OR ggt
1,740
#8 "lipid metabolism" OR "glucose metabolism" OR "fasting glucose" OR "homa ir" OR hba1c
29,149
#9 weight OR "body composition" OR "waist circumference" OR "body mass index" OR bmi OR "fat mass"
149,666
#10 "cardiorespiratory fitness" OR "physical fitness" OR "exercise test*" OR "fitness test*" OR "respirat* test*"
18,226
#11 #4 OR #5 OR #6 OR #7 OR #8 OR #9 OR #10
191,810
#12 #3 AND #11
563 (89 Reviews, 17 Cochrane protocols, 457 Trials)

Search date: March 13, 2020

PubMed Advanced search

#1 "non-alcoholic fatty liver disease"[mh] OR "non alcoholic fatty liver"[tw] OR "nonalcoholic fatty liver"[tw] OR nafld[tw] OR nash[tw] OR steatohepatitis[tw] OR steatitis[tw] OR steatosis[tw] OR "liver cirrhosis"[mh] OR "liver cirrhosis"[tw] OR "hepatic cirrhosis"[tw] OR "liver fibrosis"[tw] OR "hepatic fibrosis"[tw]

140,675

#2 exercise[mh] OR sports[mh] OR "physical activity"[tw] OR "physical activities"[tw] OR exercis*[tw] OR sport*[tw] OR gym[tw] OR gyms[tw] OR gymnastic*[tw] OR aerobic*[tw] OR training[tw] OR hiit[tw] OR walk*[tw] OR jogging[tw] OR jogger*[tw] OR running[tw] OR runner*[tw] OR marathon[tw] OR swim*[tw] OR climb*[tw] OR athletic*[tw] OR bicycl*[tw] OR football[tw] OR soccer[tw] OR baseball[tw] OR basketball[tw] OR volleyball[tw] OR skiing[tw] OR skating[tw] OR hockey[tw] OR "weight lifting"[tw] OR weightlift*[tw]

1,158,578

#3 PNPLA3[tw] OR TM6SF2[tw] OR MBOAT7[tw] OR GCKR[tw] OR SAMM50[tw] OR MnSOD[tw] OR MnSOD2[tw] OR PEMT[tw] OR LEPR[tw] OR PPP1R3B[tw] OR HSD17B13[tw]

8,503

#4 "randomized controlled trial"[pt] OR "randomized controlled trials as topic"[mh] OR "randomized controlled"[tw] OR "randomised controlled"[tw] OR "randomized control"[tw] OR "randomised control"[tw] OR "random control"[tw] OR "randomized trial"[tw] OR "randomised trial"[tw] OR "controlled trial"[tw] OR "randomized trials"[tw] OR "randomised trials"[tw] OR "controlled trials"[tw] OR "randomized study"[tw] OR "randomized studies"[tw] OR "randomised study"[tw] OR "randomised studies"[tw] OR "controlled study"[tw] OR "controlled studies"[tw] OR rct[tw] OR rcts[tw] OR random*[tw]

1,388,025

#5 #1 AND #2 AND #3 AND #4

1

#6 #5 AND english[la]

1

Scopus Advanced search

#1 TITLE-ABS-KEY("non alcoholic fatty liver*" OR "nonalcoholic fatty liver*" OR nafld OR nash OR steatohepatitis OR steatitis OR steatosis OR "liver cirrhosis" OR "hepatic cirrhosis" OR "liver fibrosis" OR "hepatic fibrosis")

230,241

#2 TITLE-ABS-KEY("physical activit*" OR exercis* OR sport* OR gym* OR aerobic* OR training OR hiit OR walk* OR jog* OR run* OR marathon OR swim* OR climb* OR athletic* OR bicycl* OR football OR soccer OR baseball OR basketball OR volleyball OR skiing OR skating OR hockey OR "weight lifting" OR weightlift*)

3,450,512

#3 TITLE-ABS-KEY(PNPLA3 OR TM6SF2 OR MBOAT7 OR GCKR OR SAMM50 OR MnSOD OR MnSOD2 OR PEMT OR LEPR OR PPP1R3B OR HSD17B13)

7,403

#4 TITLE-ABS-KEY("random* control*" OR "random* trial*" OR "control* trial*" OR "random* stud*" OR "control* stud*" OR rct OR rcts)

6,637,334

#5 #1 AND #2 AND #3 AND #4

10

#6 #5 AND LANGUAGE(english)

10

Web of Science Advanced search

#1 TS=("non alcoholic fatty liver*" OR "nonalcoholic fatty liver*" OR nafld OR nash OR steatohepatitis OR steatitis OR steatosis OR "liver cirrhosis" OR "hepatic cirrhosis" OR "liver fibrosis" OR "hepatic fibrosis")
118,982
#2 TS=("physical activit*" OR exercis* OR sport* OR gym* OR aerobic* OR training OR hiit OR walk* OR jog* OR run* OR marathon OR swim* OR climb* OR athletic* OR bicycl* OR football OR soccer OR baseball OR basketball OR volleyball OR skiing OR skating OR hockey OR "weight lifting" OR weightlift*)
2,097,027
#3 TS=(PNPLA3 OR TM6SF2 OR MBOAT7 OR GCKR OR SAMM50 OR MnSOD OR MnSOD2 OR PEMT OR LEPR OR PPP1R3B OR HSD17B13)
7,983
#4 TS=("random* control*" OR "random* trial*" OR "control* trial*" OR "random* stud*" OR "control* stud*" OR rct OR rcts)
753,515
#5 #1 AND #2 AND #3 AND #4
3
#5 #4 AND LANGUAGE: (English)
2

Cochrane Database of Systematic Reviews
Issue 3 of 12, March 2020
Cochrane Central Register of Controlled Trials
Issue 3 of 12, March 2020
#1 "non alcoholic fatty liver*" OR "nonalcoholic fatty liver*" OR nafld OR nash OR steatohepatitis OR steatitis OR steatosis OR "liver cirrhosis" OR "hepatic cirrhosis" OR "liver fibrosis" OR "hepatic fibrosis"
10,758
#2 "physical activit*" OR exercis* OR sport* OR gym* OR aerobic* OR training OR hiit OR walk* OR jog* OR run* OR marathon OR swim* OR climb* OR athletic* OR bicycl* OR football OR soccer OR baseball OR basketball OR volleyball OR skiing OR skating OR hockey OR "weight lifting" OR weightlift*
197,331
#3 PNPLA3 OR TM6SF2 OR MBOAT7 OR GCKR OR SAMM50 OR MnSOD OR MnSOD2 OR PEMT OR LEPR OR PPP1R3B OR HSD17B13
139
#4 #1 AND #2 AND #3
5 (1 protocol, 4 trials)

Supplementary Figure S3-S11: Supplementary figures for Detection of bias - Funnel plots

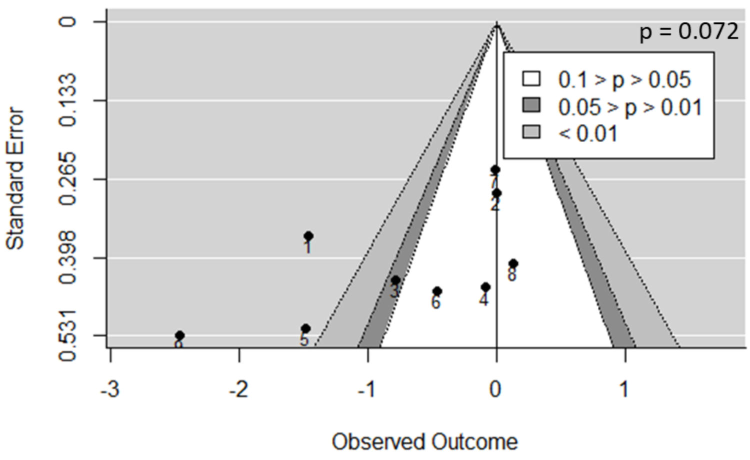

(a)

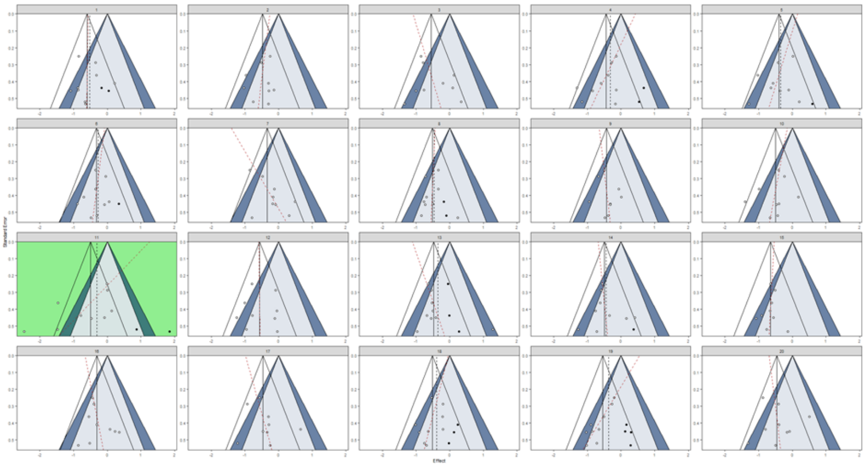

(b)

Figure S3: Detection of bias for aspartate aminotransaminase (AST). (**a**) Funnel plot of studies included in the AST meta-analysis including 95% confidence contours and significance contours at 0.05 and 0.01 levels; (**b**) Funnel plot line up. One funnel plot shows the actually observed data (green), while the other 19 funnel plots were simulated under the null hypothesis of a fixed-effect meta-analytic model. Shown are 95% confidence contours, the summary effect (vertical line), and significance contours at the 0.05 and 0.01 levels. The black dotted lines and the red lines represent the trim and fill and Egger's regression, respectively.

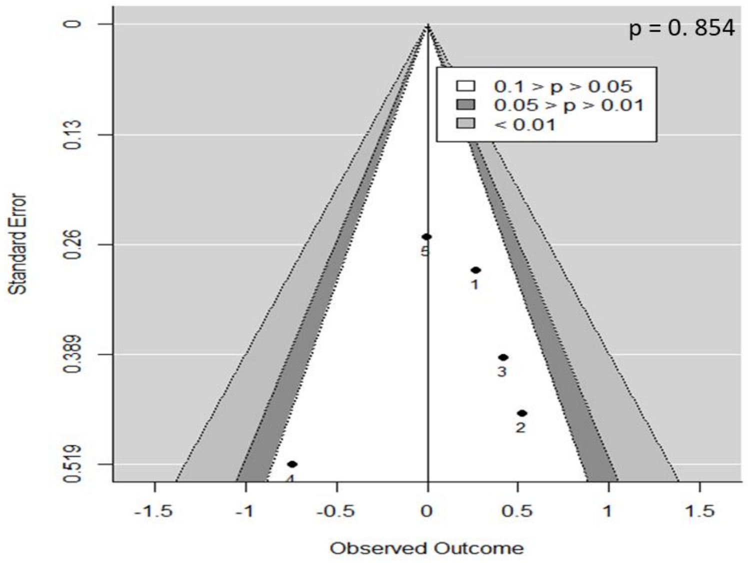

(a)

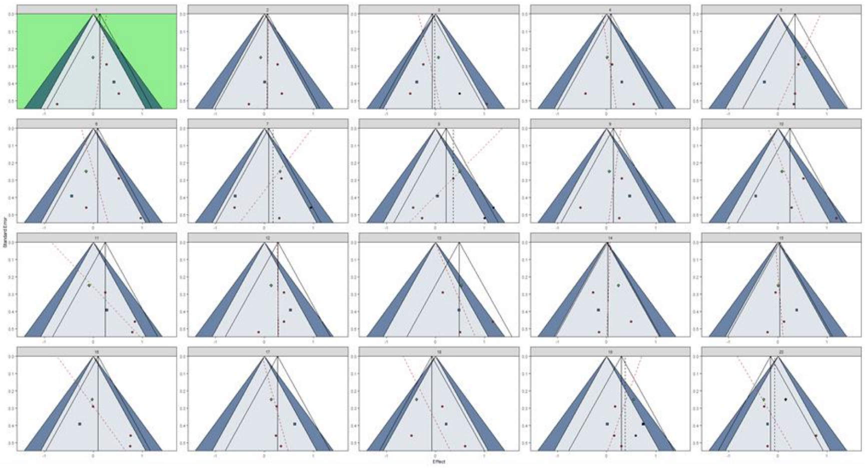

(b)

Figure S4: Detection of bias for high-density lipoprotein cholesterol (HDL-C). (**a**) Funnel plot of studies included in the HDL-C meta-analysis including 95% confidence contours and significance contours at 0.05 and 0.01 levels; (**b**) Funnel plot line up. One funnel plot shows the actually observed data (green), while the other 19 funnel plots were simulated under the null hypothesis of a fixed-effect meta-analytic model. Shown are 95% confidence contours, the summary effect (vertical line), and significance contours at the 0.05 and 0.01 levels. The black dotted lines and the red lines represent the trim and fill and Egger's regression, respectively.

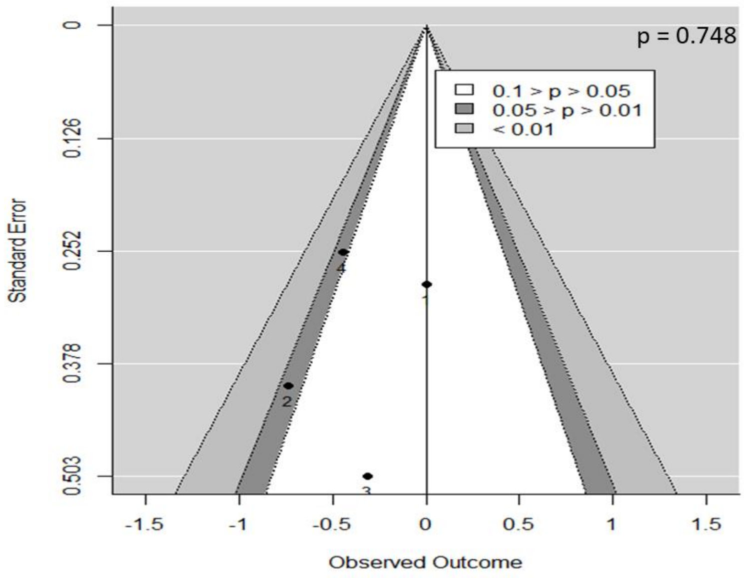

(a)

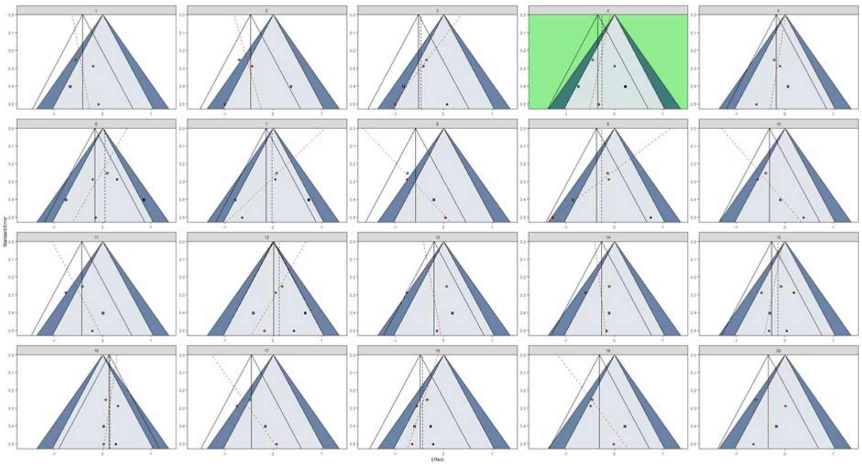

(b)

Figure S5: Detection of bias for low-density lipoprotein cholesterol (LDL-C). (**a**) Funnel plot of studies included in the LDL-C meta-analysis including 95% confidence contours and significance contours at 0.05 and 0.01 levels; (**b**) Funnel plot line up. One funnel plot shows the actually observed data (green), while the other 19 funnel plots were simulated under the null hypothesis of a fixed-effect meta-analytic model. Shown are 95% confidence contours, the summary effect (vertical line), and significance contours at the 0.05 and 0.01 levels. The black dotted lines and the red lines represent the trim and fill and Egger's regression, respectively.

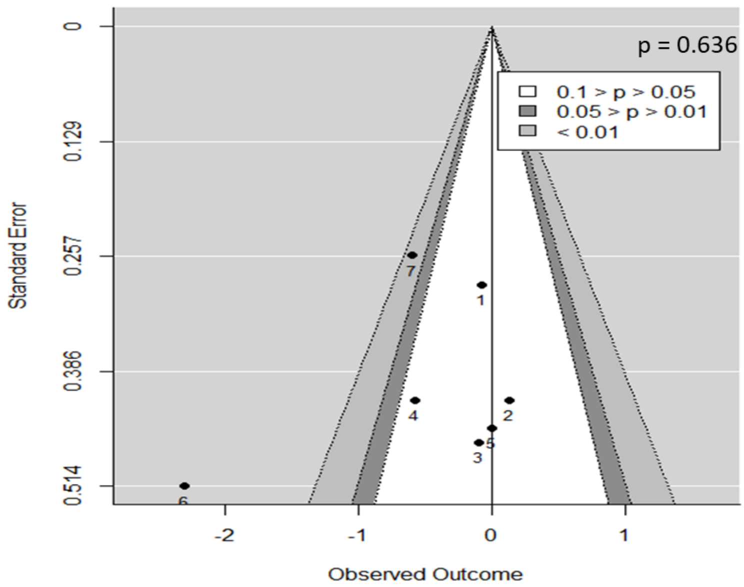

(a)

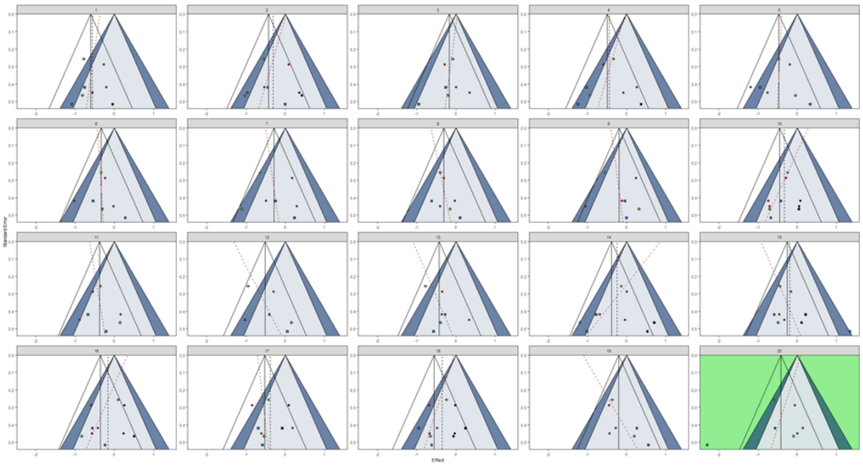

(b)

Figure S6: Detection of bias for total cholesterol (TC). (**a**) Funnel plot of studies included in the TC meta-analysis including 95% confidence contours and significance contours at 0.05 and 0.01 levels; (**b**) Funnel plot line up. One funnel plot shows the actually observed data (green), while the other 19 funnel plots were simulated under the null hypothesis of a fixed-effect meta-analytic model. Shown are 95% confidence contours, the summary effect (vertical line), and significance contours at the 0.05 and 0.01 levels. The black dotted lines and the red lines represent the trim and fill and Egger's regression, respectively.

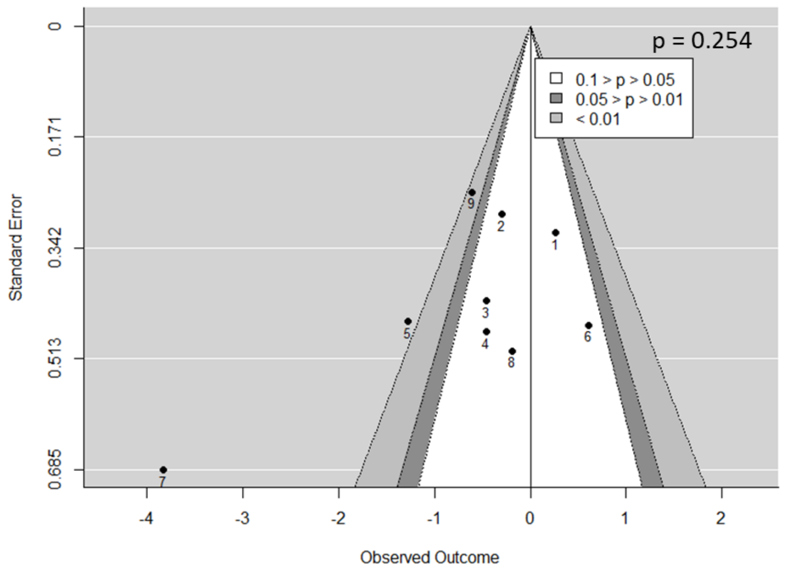

(a)

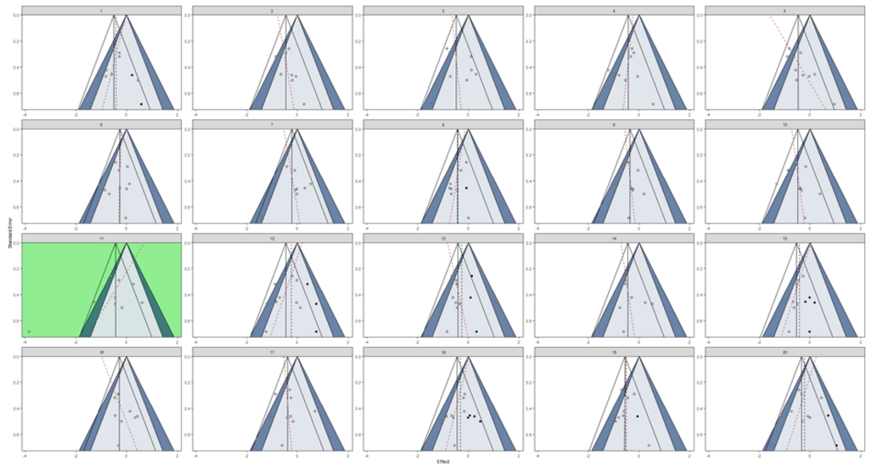

(b)

Figure S7: Detection of bias for triglyceride (TG). (**a**) Funnel plot of studies included in the TG meta-analysis including 95% confidence contours and significance contours at 0.05 and 0.01 levels; (**b**) Funnel plot line up. One funnel plot shows the actually observed data (green), while the other 19 funnel plots were simulated under the null hypothesis of a fixed-effect meta-analytic model. Shown are 95% confidence contours, the summary effect (vertical line), and significance contours at the 0.05 and 0.01 levels. The black dotted lines and the red lines represent the trim and fill and Egger's regression, respectively.

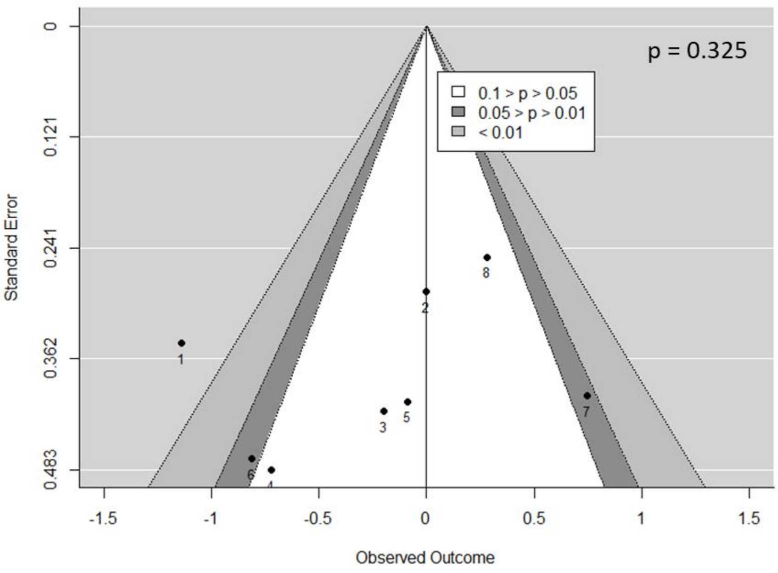

(a)

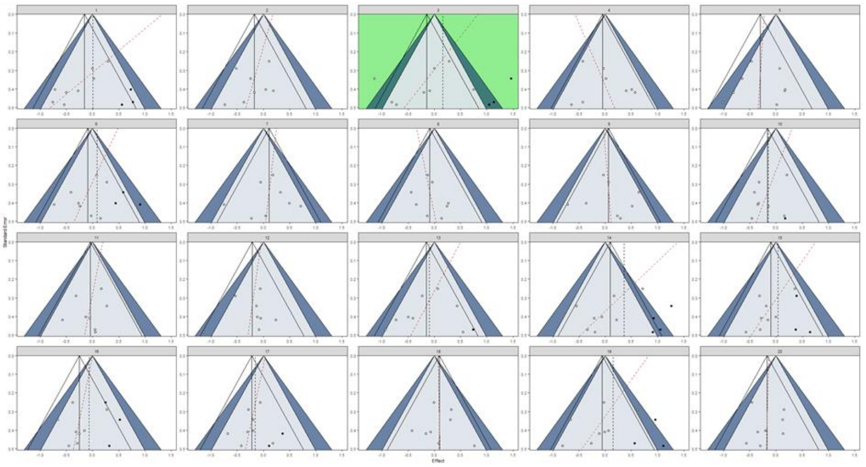

(b)

Figure S8: Detection of bias for fasting glucose. (**a**) Funnel plot of studies included in the fasting glucose meta-analysis including 95% confidence contours and significance contours at 0.05 and 0.01 levels; (**b**) Funnel plot line up. One funnel plot shows the actually observed data (green), while the other 19 funnel plots were simulated under the null hypothesis of a fixed-effect meta-analytic model. Shown are 95% confidence contours, the summary effect (vertical line), and significance contours at the 0.05 and 0.01 levels. The black dotted lines and the red lines represent the trim and fill and Egger's regression, respectively.

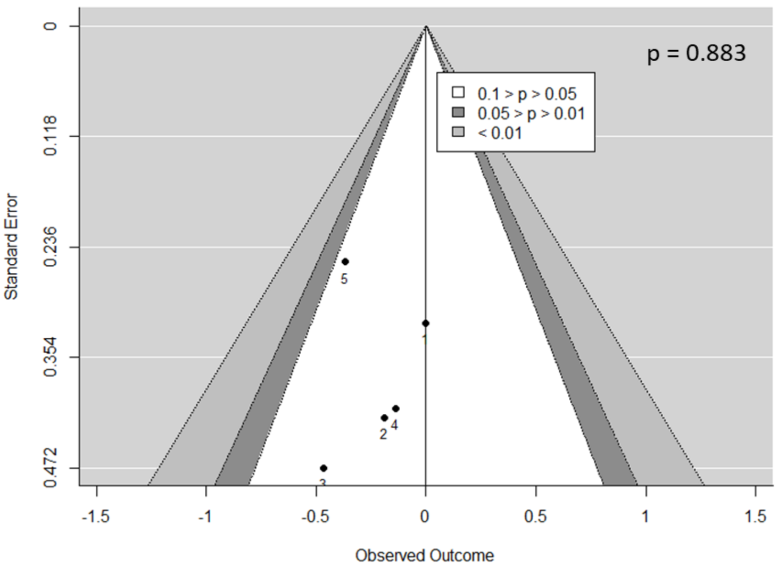

(**a**)

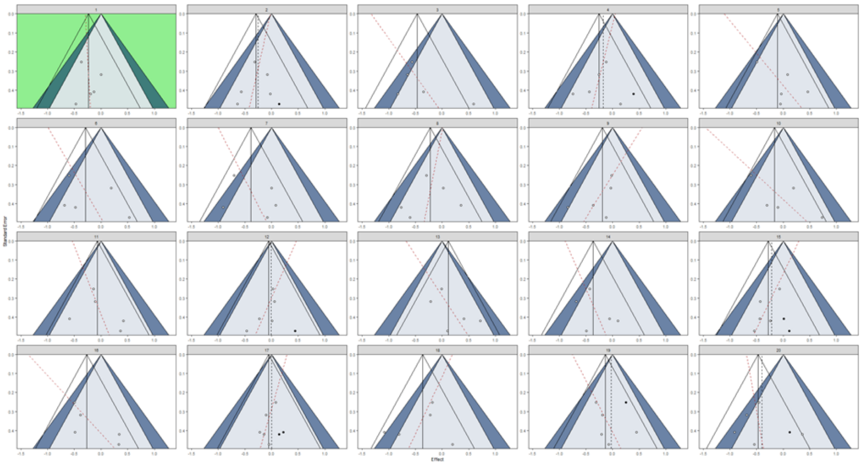

(**b**)

Figure S1: Detection of bias for glycated hemoglobin (HbA1c). (**a**) Funnel plot of studies included in the HbA1c meta-analysis including 95% confidence contours and significance contours at 0.05 and 0.01 levels; (**b**) Funnel plot line up. One funnel plot shows the actually observed data (green), while the other 19 funnel plots were simulated under the null hypothesis of a fixed-effect meta-analytic model. Shown are 95% confidence contours, the summary effect (vertical line), and significance contours at the 0.05 and 0.01 levels. The black dotted lines and the red lines represent the trim and fill and Egger's regression, respectively.

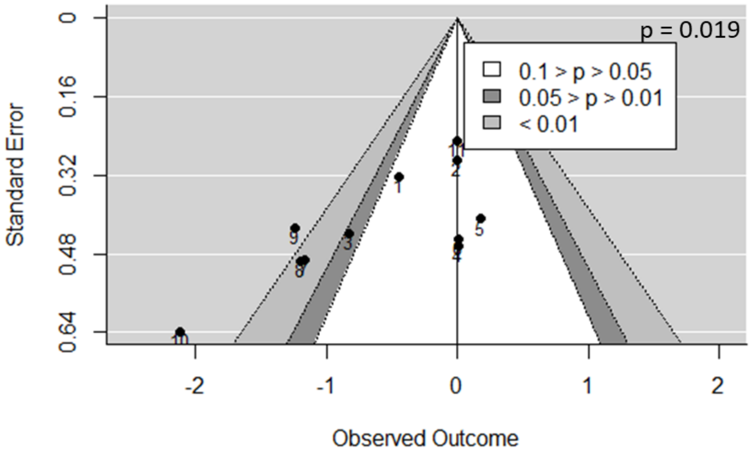

(a)

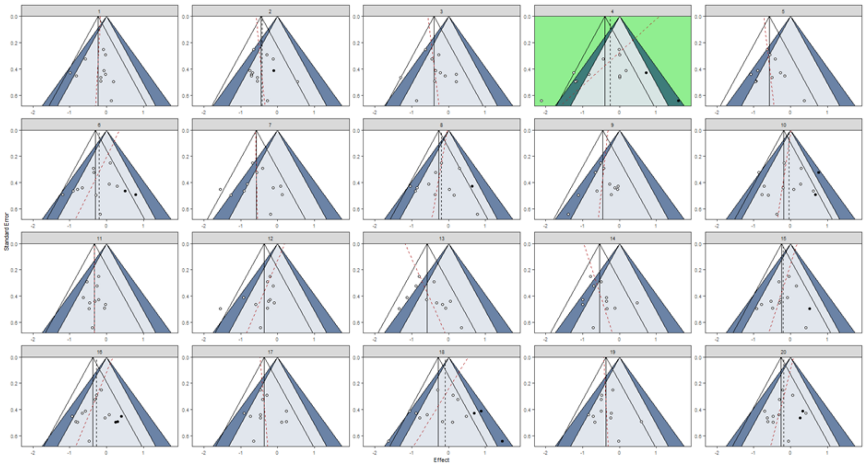

(b)

Figure S10: Detection of bias for alanine aminotransaminase (ALT). (**a**) Funnel plot of studies included in the ALT meta-analysis including 95% confidence contours and significance contours at 0.05 and 0.01 levels; (**b**) Funnel plot line up. One funnel plot shows the actually observed data (green), while the other 19 funnel plots were simulated under the null hypothesis of a fixed-effect meta-analytic model. Shown are 95% confidence contours, the summary effect (vertical line), and significance contours at the 0.05 and 0.01 levels. The black dotted lines and the red lines represent the trim and fill and Egger's regression, respectively.

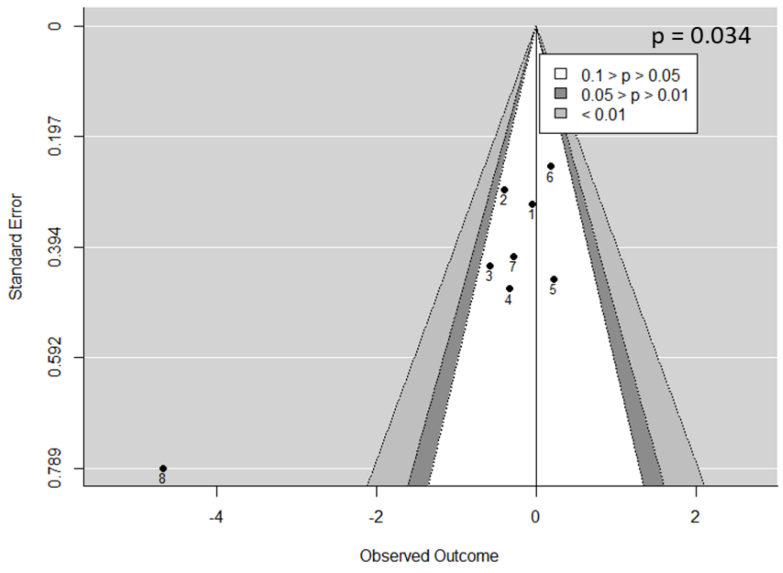

(a)

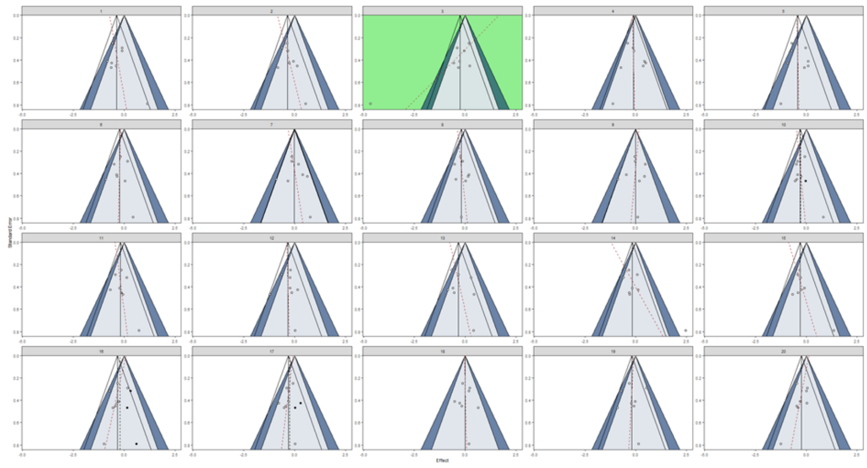

(b)

Figure S11: Detection of bias for fasting insulin. (**a**) Funnel plot of studies included in the fasting insulin meta-analysis including 95% confidence contours and significance contours at 0.05 and 0.01 levels; (**b**) Funnel plot line up. One funnel plot shows the actually observed data (green), while the other 19 funnel plots were simulated under the null hypothesis of a fixed-effect meta-analytic model. Shown are 95% confidence contours, the summary effect (vertical line), and significance contours at the 0.05 and 0.01 levels. The black dotted lines and the red lines represent the trim and fill and Egger's regression, respectively.

Supplementary Figure S12 – S16: Supplementary figures for Outlier detection

```
Identified outliers (fixed-effect model)
----------------------------------------
"Cheng et al. 2017, China", "Shojaee-Moradie et al. 2016, UK"

Results with outliers removed
-----------------------------
                                    SMD             95%-CI %W(fixed) %W(random) exclude
Cheng et al. 2017, China        -1.4637 [-2.1723; -0.7550]       0.0        0.0       *
Cuthbertson et al. 2016, UK      0.0000 [-0.5658;  0.5658]      22.8       19.6
Hallsworth et al. 2015, UK      -0.7871 [-1.6426;  0.0683]      10.0       12.2
Pugh et al. 2014, UK            -0.0896 [-0.9708;  0.7917]       9.4       11.7
Shamsoddini et al. 2015, Iran   -1.4833 [-2.4989; -0.4677]       7.1        9.6
Shamsoddini et al. 2015, Iran   -0.4683 [-1.3596;  0.4231]       9.2       11.5
Zelberg-Sagi et al. 2014, Israel -0.0107 [-0.5010;  0.4795]      30.3       22.1
Houghton et al. 2017, UK         0.1294 [-0.6718;  0.9305]      11.4       13.3
Shojaee-Moradie et al. 2016, UK -2.4667 [-3.5074; -1.4261]       0.0        0.0       *

Number of studies combined: k = 7

                         SMD            95%-CI     z p-value
Fixed effect model   -0.2231 [-0.4930; 0.0469] -1.62  0.1053
Random effects model -0.2876 [-0.6527; 0.0775] -1.54  0.1226

Quantifying heterogeneity:
 tau^2 = 0.0941 [0.0000; 1.2553]; tau = 0.3068 [0.0000; 1.1204];
 I^2 = 40.2% [0.0%; 74.8%]; H = 1.29 [1.00; 1.99]

Test of heterogeneity:
     Q d.f. p-value
 10.03    6  0.1236

Details on meta-analytical method:
- Inverse variance method
- DerSimonian-Laird estimator for tau^2
- Jackson method for confidence interval of tau^2 and tau
- Hedges' g (bias corrected standardised mean difference)
```

(**a**)

```
Identified outliers (random-effects model)
------------------------------------------
"Shojaee-Moradie et al. 2016, UK"

Results with outliers removed
-----------------------------
                                    SMD             95%-CI %W(fixed) %W(random) exclude
Cheng et al. 2017, China        -1.4637 [-2.1723; -0.7550]      12.7       13.2
Cuthbertson et al. 2016, UK      0.0000 [-0.5658;  0.5658]      19.9       15.1
Hallsworth et al. 2015, UK      -0.7871 [-1.6426;  0.0683]       8.7       11.5
Pugh et al. 2014, UK            -0.0896 [-0.9708;  0.7917]       8.2       11.2
Shamsoddini et al. 2015, Iran   -1.4833 [-2.4989; -0.4677]       6.2        9.8
Shamsoddini et al. 2015, Iran   -0.4683 [-1.3596;  0.4231]       8.0       11.1
Zelberg-Sagi et al. 2014, Israel -0.0107 [-0.5010;  0.4795]      26.5       16.0
Houghton et al. 2017, UK         0.1294 [-0.6718;  0.9305]       9.9       12.1
Shojaee-Moradie et al. 2016, UK -2.4667 [-3.5074; -1.4261]       0.0        0.0       *

Number of studies combined: k = 8

                         SMD             95%-CI     z p-value
Fixed effect model   -0.3803 [-0.6325; -0.1280] -2.95  0.0031
Random effects model -0.4776 [-0.9247; -0.0305] -2.09  0.0363

Quantifying heterogeneity:
 tau^2 = 0.2624 [0.0311; 1.6087]; tau = 0.5122 [0.1762; 1.2684];
 I^2 = 65.5% [26.7%; 83.8%]; H = 1.70 [1.17; 2.48]

Test of heterogeneity:
     Q d.f. p-value
 20.31    7  0.0049

Details on meta-analytical method:
- Inverse variance method
- DerSimonian-Laird estimator for tau^2
- Jackson method for confidence interval of tau^2 and tau
- Hedges' g (bias corrected standardised mean difference)
```

(**b**)

Figure S12: Outlier detection with (**a**) fixed effect and (**b**) random effects models for aspartate aminotransaminase (AST).

```
Identified outliers (fixed-effect model)
----------------------------------------
"Shojaee-Moradie et al. 2016, UK"

Results with outliers removed
-----------------------------
                                    SMD             95%-CI %W(fixed) %W(random) exclude
Cuthbertson et al. 2016, UK     -0.0729 [-0.6389;  0.4931]      24.8       24.8
Hallsworth et al. 2015, UK       0.1369 [-0.6824;  0.9562]      11.9       11.9
Hallsworth et al. 2011, UK      -0.1004 [-1.0118;  0.8110]       9.6        9.6
Houghton et al. 2017, UK        -0.5741 [-1.3938;  0.2455]      11.8       11.8
Pugh et al. 2014, UK             0.0000 [-0.8807;  0.8807]      10.3       10.3
Shojaee-Moradie et al. 2016, UK -2.2998 [-3.3082; -1.2915]       0.0        0.0       *
Zelberg-Sagi et al. 2014, Israel -0.6013 [-1.1032; -0.0994]      31.6       31.6

Number of studies combined: k = 6

                         SMD            95%-CI     z p-value
Fixed effect model   -0.2695 [-0.5517; 0.0126] -1.87  0.0611
Random effects model -0.2695 [-0.5517; 0.0126] -1.87  0.0611

Quantifying heterogeneity:
 tau^2 = 0 [0.0000; 0.4731]; tau = 0 [0.0000; 0.6878];
 I^2 = 0.0% [0.0%; 69.1%]; H = 1.00 [1.00; 1.80]

Test of heterogeneity:
    Q d.f. p-value
 4.11    5  0.5337

Details on meta-analytical method:
- Inverse variance method
- DerSimonian-Laird estimator for tau^2
- Jackson method for confidence interval of tau^2 and tau
- Hedges' g (bias corrected standardised mean difference)
```

(**a**)

```
Identified outliers (random-effects model)
------------------------------------------
"Shojaee-Moradie et al. 2016, UK"

Results with outliers removed
-----------------------------
                                    SMD             95%-CI %W(fixed) %W(random) exclude
Cuthbertson et al. 2016, UK     -0.0729 [-0.6389;  0.4931]      24.8       24.8
Hallsworth et al. 2015, UK       0.1369 [-0.6824;  0.9562]      11.9       11.9
Hallsworth et al. 2011, UK      -0.1004 [-1.0118;  0.8110]       9.6        9.6
Houghton et al. 2017, UK        -0.5741 [-1.3938;  0.2455]      11.8       11.8
Pugh et al. 2014, UK             0.0000 [-0.8807;  0.8807]      10.3       10.3
Shojaee-Moradie et al. 2016, UK -2.2998 [-3.3082; -1.2915]       0.0        0.0       *
Zelberg-Sagi et al. 2014, Israel -0.6013 [-1.1032; -0.0994]      31.6       31.6

Number of studies combined: k = 6

                         SMD            95%-CI     z p-value
Fixed effect model   -0.2695 [-0.5517; 0.0126] -1.87  0.0611
Random effects model -0.2695 [-0.5517; 0.0126] -1.87  0.0611

Quantifying heterogeneity:
 tau^2 = 0 [0.0000; 0.4731]; tau = 0 [0.0000; 0.6878];
 I^2 = 0.0% [0.0%; 69.1%]; H = 1.00 [1.00; 1.80]

Test of heterogeneity:
    Q d.f. p-value
 4.11    5  0.5337

Details on meta-analytical method:
- Inverse variance method
- DerSimonian-Laird estimator for tau^2
- Jackson method for confidence interval of tau^2 and tau
- Hedges' g (bias corrected standardised mean difference)
```

(**b**)

Figure S13: Outlier detection with (**a**) fixed effect and (**b**) random effects models for total cholesterol (TC).

```
Identified outliers (fixed-effect model)
----------------------------------------
"Shojaee-Moradie et al. 2016, UK"

Results with outliers removed
-----------------------------
                                     SMD             95%-CI %W(fixed) %W(random) exclude
Cheng et al. 2017, China          0.2683 [-0.3577;  0.8943]      16.2       15.1
Cuthbertson et al. 2016, UK      -0.3002 [-0.8693;  0.2689]      19.6       16.3
Hallsworth et al. 2015, UK       -0.4562 [-1.2870;  0.3747]       9.2       11.2
Hallsworth et al. 2011, UK       -0.4559 [-1.3811;  0.4693]       7.4        9.9
Houghton et al. 2017, UK         -1.2785 [-2.1711; -0.3859]       8.0       10.3
Pugh et al. 2014, UK              0.6042 [-0.2995;  1.5080]       7.8       10.1
Shojaee-Moradie et al. 2016, UK  -3.8331 [-5.1747; -2.4914]       0.0        0.0       *
Sullivan et al. 2012, USA        -0.1880 [-1.1705;  0.7944]       6.6        9.1
Zelberg-Sagi et al. 2014, Israel -0.6137 [-1.1161; -0.1113]      25.2       17.9

Number of studies combined: k = 8

                         SMD              95%-CI     z p-value
Fixed effect model   -0.3131 [-0.5652; -0.0610] -2.43  0.0149
Random effects model -0.3024 [-0.6658;  0.0610] -1.63  0.1029

Quantifying heterogeneity:
 tau^2 = 0.1261 [0.0000; 1.0747]; tau = 0.3551 [0.0000; 1.0367];
 I^2 = 47.8% [0.0%; 76.8%]; H = 1.38 [1.00; 2.08]

Test of heterogeneity:
     Q d.f. p-value
 13.41    7  0.0627

Details on meta-analytical method:
- Inverse variance method
- DerSimonian-Laird estimator for tau^2
- Jackson method for confidence interval of tau^2 and tau
- Hedges' g (bias corrected standardised mean difference)
```

(**a**)

```
Identified outliers (random-effects model)
------------------------------------------
"Shojaee-Moradie et al. 2016, UK"

Results with outliers removed
-----------------------------
                                     SMD             95%-CI %W(fixed) %W(random) exclude
Cheng et al. 2017, China          0.2683 [-0.3577;  0.8943]      16.2       15.1
Cuthbertson et al. 2016, UK      -0.3002 [-0.8693;  0.2689]      19.6       16.3
Hallsworth et al. 2015, UK       -0.4562 [-1.2870;  0.3747]       9.2       11.2
Hallsworth et al. 2011, UK       -0.4559 [-1.3811;  0.4693]       7.4        9.9
Houghton et al. 2017, UK         -1.2785 [-2.1711; -0.3859]       8.0       10.3
Pugh et al. 2014, UK              0.6042 [-0.2995;  1.5080]       7.8       10.1
Shojaee-Moradie et al. 2016, UK  -3.8331 [-5.1747; -2.4914]       0.0        0.0       *
Sullivan et al. 2012, USA        -0.1880 [-1.1705;  0.7944]       6.6        9.1
Zelberg-Sagi et al. 2014, Israel -0.6137 [-1.1161; -0.1113]      25.2       17.9

Number of studies combined: k = 8

                         SMD              95%-CI     z p-value
Fixed effect model   -0.3131 [-0.5652; -0.0610] -2.43  0.0149
Random effects model -0.3024 [-0.6658;  0.0610] -1.63  0.1029

Quantifying heterogeneity:
 tau^2 = 0.1261 [0.0000; 1.0747]; tau = 0.3551 [0.0000; 1.0367];
 I^2 = 47.8% [0.0%; 76.8%]; H = 1.38 [1.00; 2.08]

Test of heterogeneity:
     Q d.f. p-value
 13.41    7  0.0627

Details on meta-analytical method:
- Inverse variance method
- DerSimonian-Laird estimator for tau^2
- Jackson method for confidence interval of tau^2 and tau
- Hedges' g (bias corrected standardised mean difference)
```

(**b**)

Figure S14: Outlier detection with (**a**) fixed effect and (**b**) random effects models for triglyceride (TG)

```
Identified outliers (fixed-effect model)
----------------------------------------
"Shojaee-Moradie et al. 2016, UK"

Results with outliers removed
-----------------------------
                                     SMD             95%-CI %W(fixed) %W(random) exclude
Cheng et al. 2017, China         -0.0427 [-0.6657;  0.5803]      16.9       16.9
Cuthbertson et al. 2016, UK      -0.3899 [-0.9612;  0.1815]      20.1       20.1
Hallsworth et al. 2015, UK       -0.5693 [-1.4072;  0.2686]       9.4        9.4
Hallsworth et al. 2011, UK       -0.3339 [-1.2524;  0.5846]       7.8        7.8
Pugh et al. 2014, UK              0.2223 [-0.6616;  1.1061]       8.4        8.4
Zelberg-Sagi et al. 2014, Israel  0.1896 [-0.3018;  0.6810]      27.2       27.2
Houghton et al. 2017, UK         -0.2761 [-1.0808;  0.5286]      10.1       10.1
Shojaee-Moradie et al. 2016, UK  -4.6670 [-6.2131; -3.1209]       0.0        0.0       *

Number of studies combined: k = 7

                         SMD             95%-CI     z p-value
Fixed effect model   -0.1228 [-0.3791; 0.1336] -0.94  0.3480
Random effects model -0.1228 [-0.3791; 0.1336] -0.94  0.3480

Quantifying heterogeneity:
 tau^2 = 0 [0.0000; 0.3078]; tau = 0 [0.0000; 0.5548];
 I^2 = 0.0% [0.0%; 60.8%]; H = 1.00 [1.00; 1.60]

Test of heterogeneity:
    Q d.f. p-value
 4.47    6  0.6128

Details on meta-analytical method:
- Inverse variance method
- DerSimonian-Laird estimator for tau^2
- Jackson method for confidence interval of tau^2 and tau
- Hedges' g (bias corrected standardised mean difference)
```

(**a**)

```
Identified outliers (random-effects model)
------------------------------------------
"Shojaee-Moradie et al. 2016, UK"

Results with outliers removed
-----------------------------
                                     SMD             95%-CI %W(fixed) %W(random) exclude
Cheng et al. 2017, China         -0.0427 [-0.6657;  0.5803]      16.9       16.9
Cuthbertson et al. 2016, UK      -0.3899 [-0.9612;  0.1815]      20.1       20.1
Hallsworth et al. 2015, UK       -0.5693 [-1.4072;  0.2686]       9.4        9.4
Hallsworth et al. 2011, UK       -0.3339 [-1.2524;  0.5846]       7.8        7.8
Pugh et al. 2014, UK              0.2223 [-0.6616;  1.1061]       8.4        8.4
Zelberg-Sagi et al. 2014, Israel  0.1896 [-0.3018;  0.6810]      27.2       27.2
Houghton et al. 2017, UK         -0.2761 [-1.0808;  0.5286]      10.1       10.1
Shojaee-Moradie et al. 2016, UK  -4.6670 [-6.2131; -3.1209]       0.0        0.0       *

Number of studies combined: k = 7

                         SMD             95%-CI     z p-value
Fixed effect model   -0.1228 [-0.3791; 0.1336] -0.94  0.3480
Random effects model -0.1228 [-0.3791; 0.1336] -0.94  0.3480

Quantifying heterogeneity:
 tau^2 = 0 [0.0000; 0.3078]; tau = 0 [0.0000; 0.5548];
 I^2 = 0.0% [0.0%; 60.8%]; H = 1.00 [1.00; 1.60]

Test of heterogeneity:
    Q d.f. p-value
 4.47    6  0.6128

Details on meta-analytical method:
- Inverse variance method
- DerSimonian-Laird estimator for tau^2
- Jackson method for confidence interval of tau^2 and tau
- Hedges' g (bias corrected standardised mean difference)
```

(**b**)

Figure S2: Outlier detection with (**a**) fixed effect and (**b**) random effects models for fasting insulin.

```
Identified outliers (fixed-effect model)
----------------------------------------
"Sullivan et al. 2012, USA"

Results with outliers removed
-----------------------------
                                     SMD             95%-CI %W(fixed) %W(random) exclude
Cheng et al. 2017, China         -0.4439 [-1.0752;  0.1874]      13.4       12.2        
Cuthbertson et al. 2016, UK       0.0000 [-0.5658;  0.5658]      16.7       13.3        
Hallsworth et al. 2015, UK       -0.8267 [-1.6859;  0.0325]       7.2        8.9        
Hallsworth et al. 2011, UK        0.0075 [-0.9033;  0.9182]       6.4        8.3        
Houghton et al. 2017, UK          0.1760 [-0.6260;  0.9780]       8.3        9.6        
Pugh et al. 2014, UK              0.0038 [-0.8770;  0.8845]       6.9        8.6        
Shamsoddini et al. 2015, Iran    -1.1687 [-2.1340; -0.2035]       5.7        7.7        
Shamsoddini et al. 2015, Iran    -1.1995 [-2.1692; -0.2298]       5.7        7.7        
Shojaee-Moradie et al. 2016, UK  -1.2443 [-2.0841; -0.4046]       7.6        9.1        
Sullivan et al. 2012, USA        -2.1195 [-3.3742; -0.8649]       0.0        0.0       *
Zelberg-Sagi et al. 2014, Israel -0.0072 [-0.4974;  0.4830]      22.2       14.7        

Number of studies combined: k = 10

                         SMD             95%-CI     z p-value
Fixed effect model   -0.3344 [-0.5653; -0.1035] -2.84  0.0045
Random effects model -0.4059 [-0.7404; -0.0714] -2.38  0.0174

Quantifying heterogeneity:
 tau^2 = 0.1358 [0.0000; 0.8949]; tau = 0.3685 [0.0000; 0.9460];
 I^2 = 48.7% [0.0%; 75.2%]; H = 1.40 [1.00; 2.01]

Test of heterogeneity:
     Q d.f. p-value
 17.53    9  0.0410

Details on meta-analytical method:
- Inverse variance method
- DerSimonian-Laird estimator for tau^2
- Jackson method for confidence interval of tau^2 and tau
- Hedges' g (bias corrected standardised mean difference)
```

Figure S16: Outlier detection with fixed effect models for alanine aminotransaminase (ALT)

```
Identified outliers (fixed-effect model)
----------------------------------------
"Cheng et al. 2017, China"

Results with outliers removed
-----------------------------
                                     SMD             95%-CI %W(fixed) %W(random) exclude
Cheng et al. 2017, China         -1.1384 [-1.8145; -0.4623]       0.0        0.0       *
Cuthbertson et al. 2016, UK       0.0000 [-0.5658;  0.5658]      22.0       19.0        
Hallsworth et al. 2015, UK       -0.2003 [-1.0209;  0.6203]      10.5       12.5        
Hallsworth et al. 2011, UK       -0.7226 [-1.6691;  0.2240]       7.9       10.3        
Houghton et al. 2017, UK         -0.0907 [-0.8913;  0.7100]      11.0       12.9        
Pugh et al. 2014, UK             -0.8142 [-1.7364;  0.1079]       8.3       10.7        
Shojaee-Moradie et al. 2016, UK   0.7464 [-0.0427;  1.5354]      11.3       13.2        
Zelberg-Sagi et al. 2014, Israel  0.2803 [-0.2125;  0.7731]      29.0       21.4        

Number of studies combined: k = 7

                         SMD            95%-CI     z p-value
Fixed effect model    0.0106 [-0.2549; 0.2761]  0.08  0.9377
Random effects model -0.0398 [-0.3995; 0.3198] -0.22  0.8281

Quantifying heterogeneity:
 tau^2 = 0.0939 [0.0000; 1.1516]; tau = 0.3064 [0.0000; 1.0731];
 I^2 = 41.1% [0.0%; 75.2%]; H = 1.30 [1.00; 2.01]

Test of heterogeneity:
     Q d.f. p-value
 10.19    6  0.1171

Details on meta-analytical method:
- Inverse variance method
- DerSimonian-Laird estimator for tau^2
- Jackson method for confidence interval of tau^2 and tau
- Hedges' g (bias corrected standardised mean difference)
```

Figure S17: Outlier detection with fixed effect model for fasting glucose.

<u>Supplementary Figure S18- S26: Supplementary figures for Influence analysis</u>

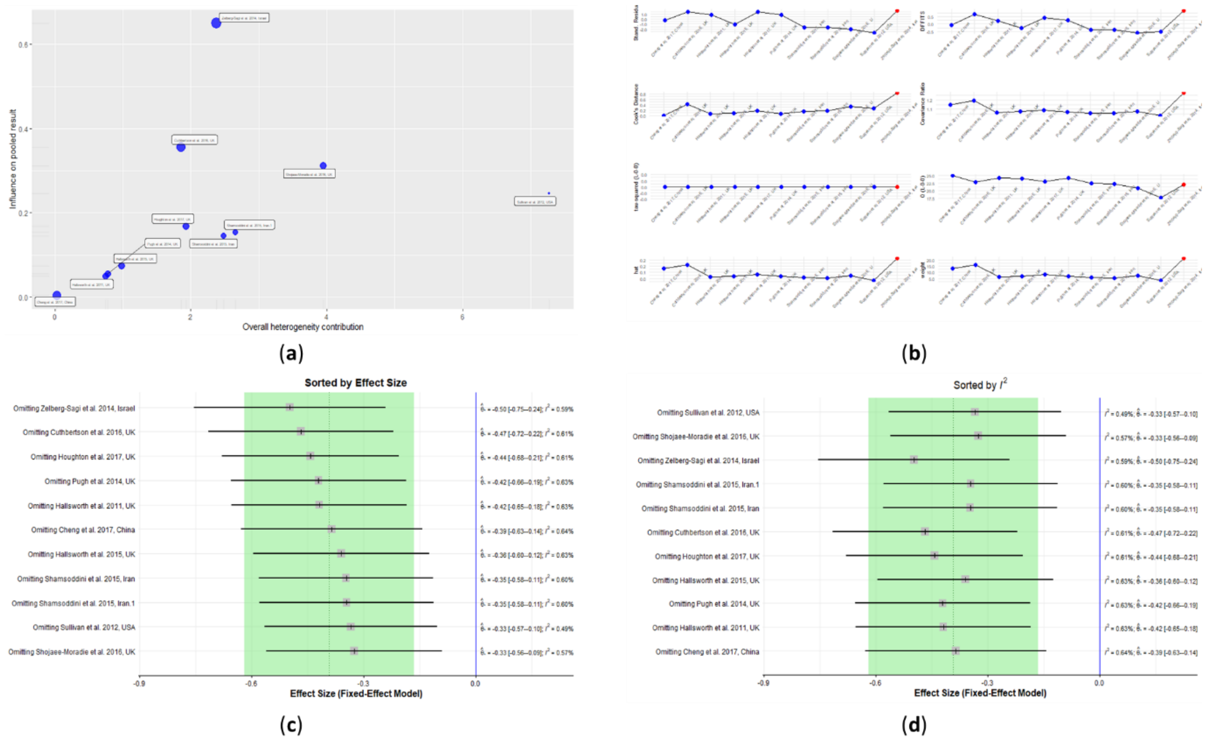

Figure S18: Influence analysis plots for alanine aminotransaminase (ALT). (**a**) Baujat plot; (**b**) Diagnostic tests for influence measures; (**c**)Leave-one-out analyses sorted by decreasing order of effect size; (**d**) Leave-one-out analyses sorted by decreasing order of heterogeneity.

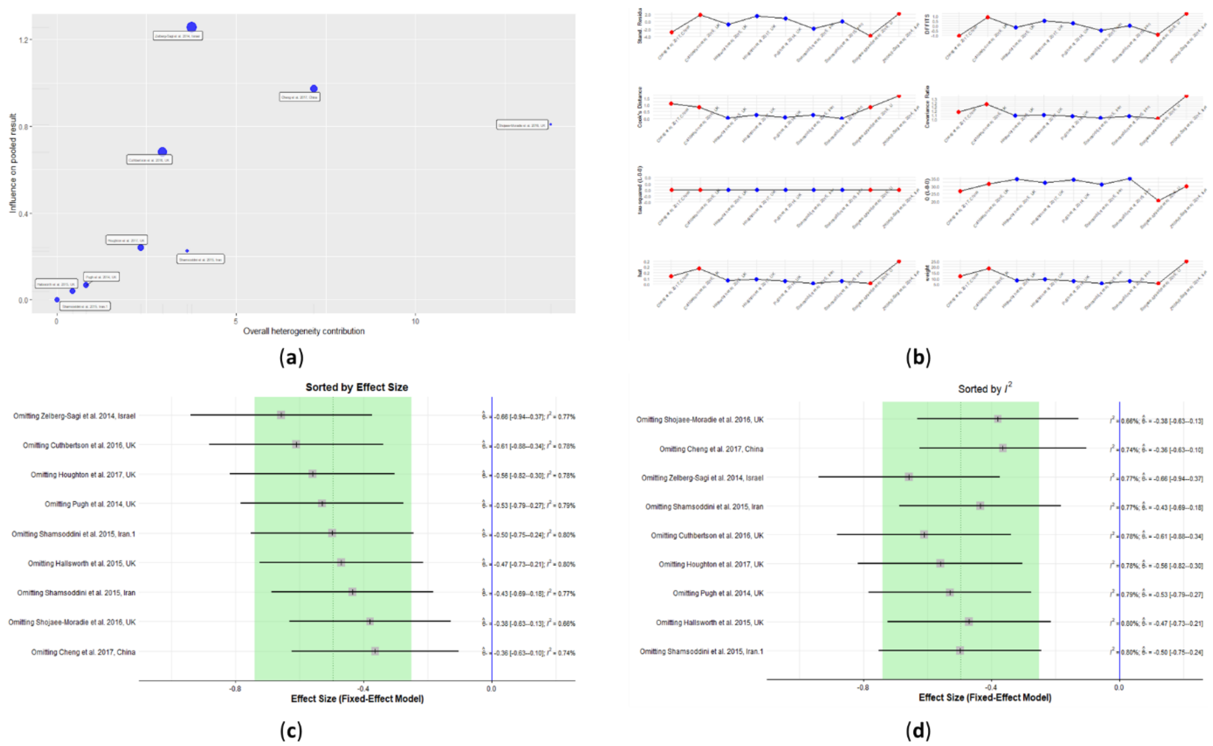

Figure S19: Influence analysis plots for aspartate aminotransaminase (AST). (**a**) Baujat plot; (**b**) Diagnostic tests for influence measures; (**c**)Leave-one-out analyses sorted by decreasing order of effect size; (**d**) Leave-one-out analyses sorted by decreasing order of heterogeneity

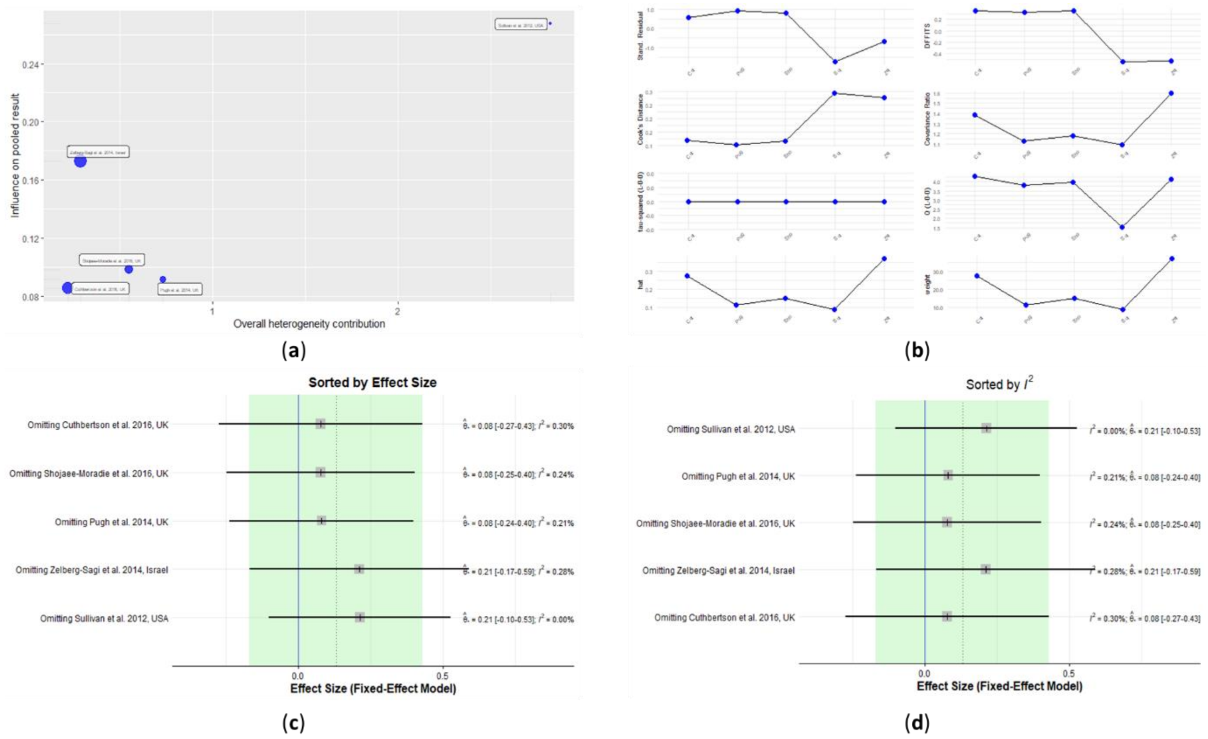

Figure S20: Influence analysis plots for high-density lipoprotein cholesterol (HDL-C). (**a**) Baujat plot; (**b**) Diagnostic tests for influence measures; (**c**)Leave-one-out analyses sorted by decreasing order of effect size; (**d**) Leave-one-out analyses sorted by decreasing order of heterogeneity

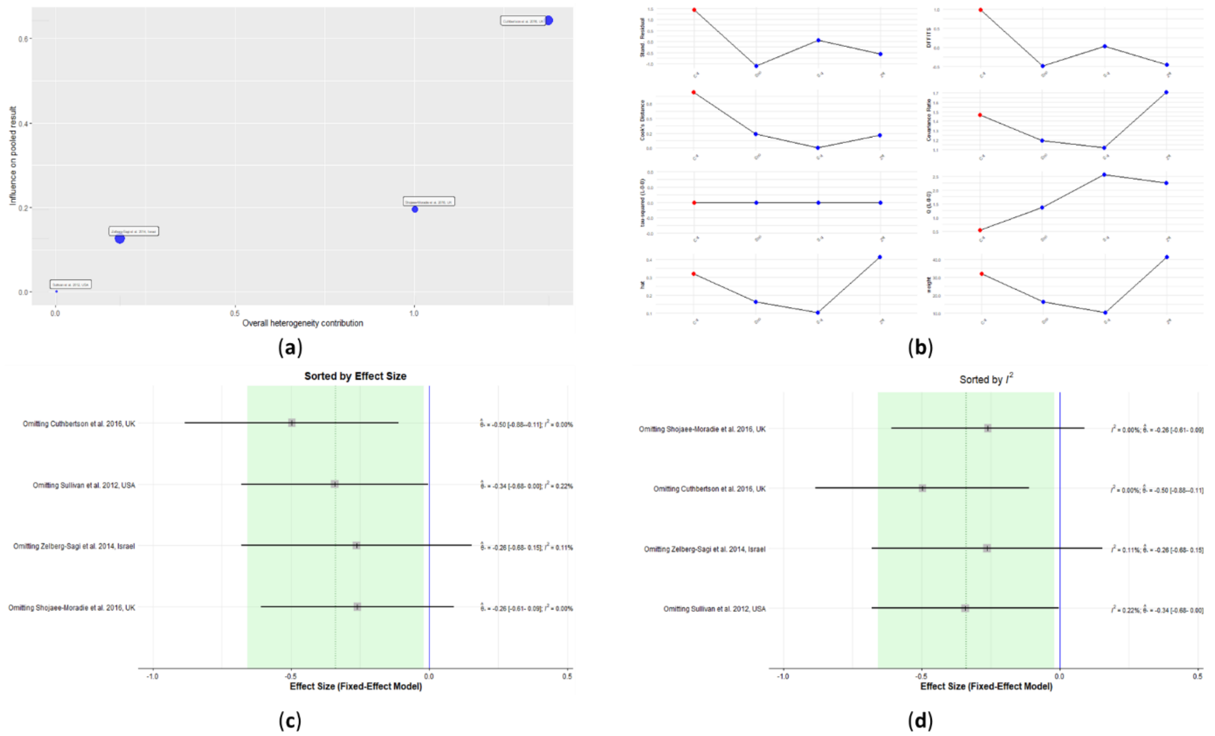

Figure S21: Influence analysis plots for low-density lipoprotein cholesterol (LDL-C). (**a**) Baujat plot; (**b**) Diagnostic tests for influence measures; (**c**)Leave-one-out analyses sorted by decreasing order of effect size; (**d**) Leave-one-out analyses sorted by decreasing order of heterogeneity.

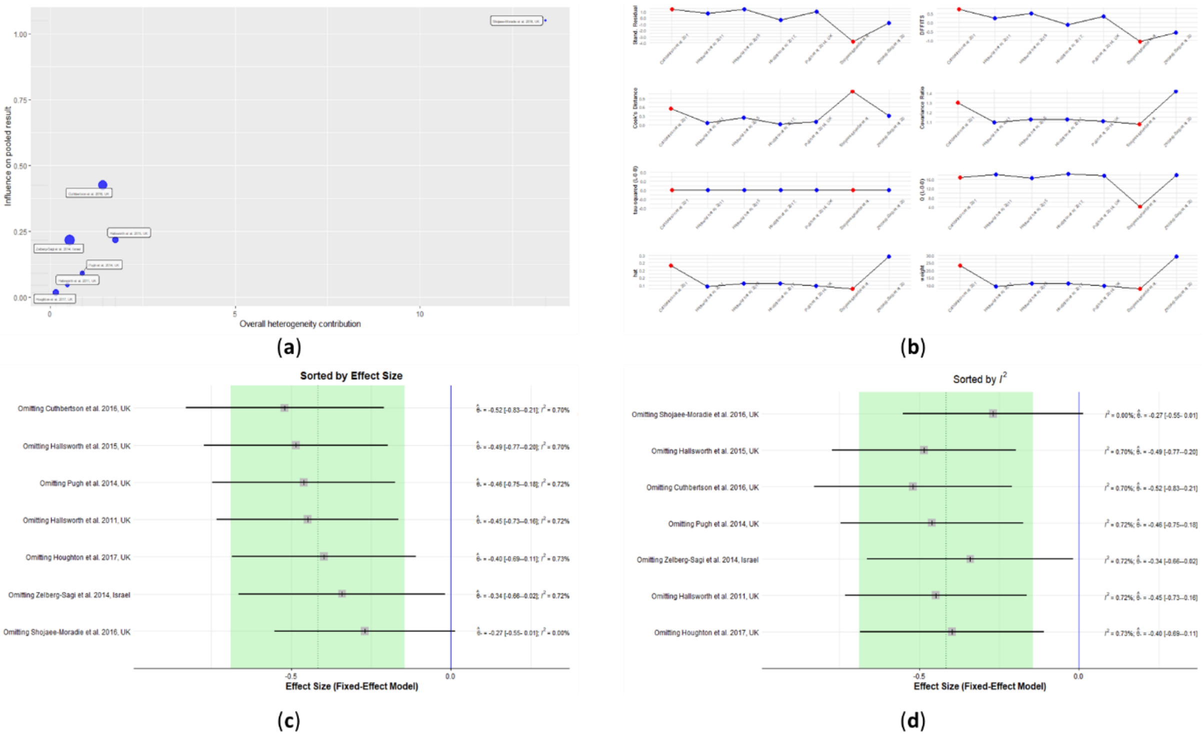

Figure S22: Influence analysis plots for total cholesterol (TC). (**a**) Baujat plot; (**b**) Diagnostic tests for influence measures; (**c**)Leave-one-out analyses sorted by decreasing order of effect size; (**d**) Leave-one-out analyses sorted by decreasing order of heterogeneity.

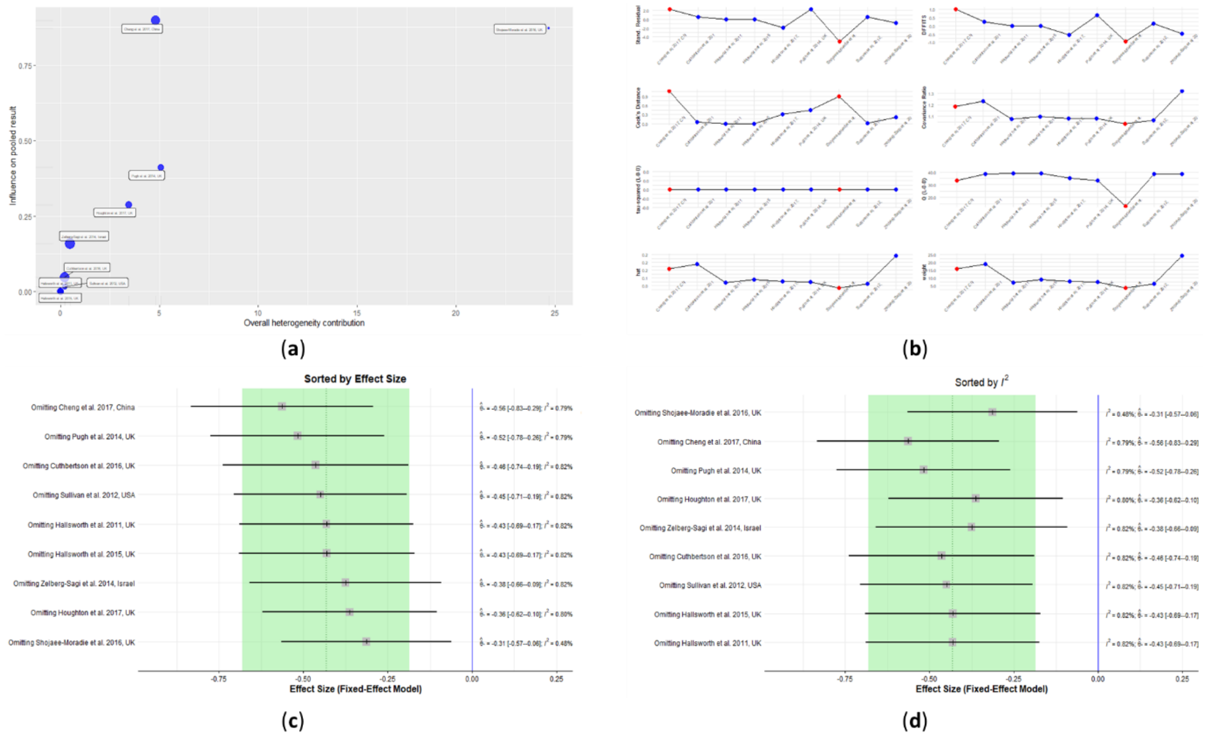

Figure S23: Influence analysis plots for triglyceride (TG). (**a**) Baujat plot; (**b**) Diagnostic tests for influence measures; (**c**)Leave-one-out analyses sorted by decreasing order of effect size; (**d**) Leave-one-out analyses sorted by decreasing order of heterogeneity.

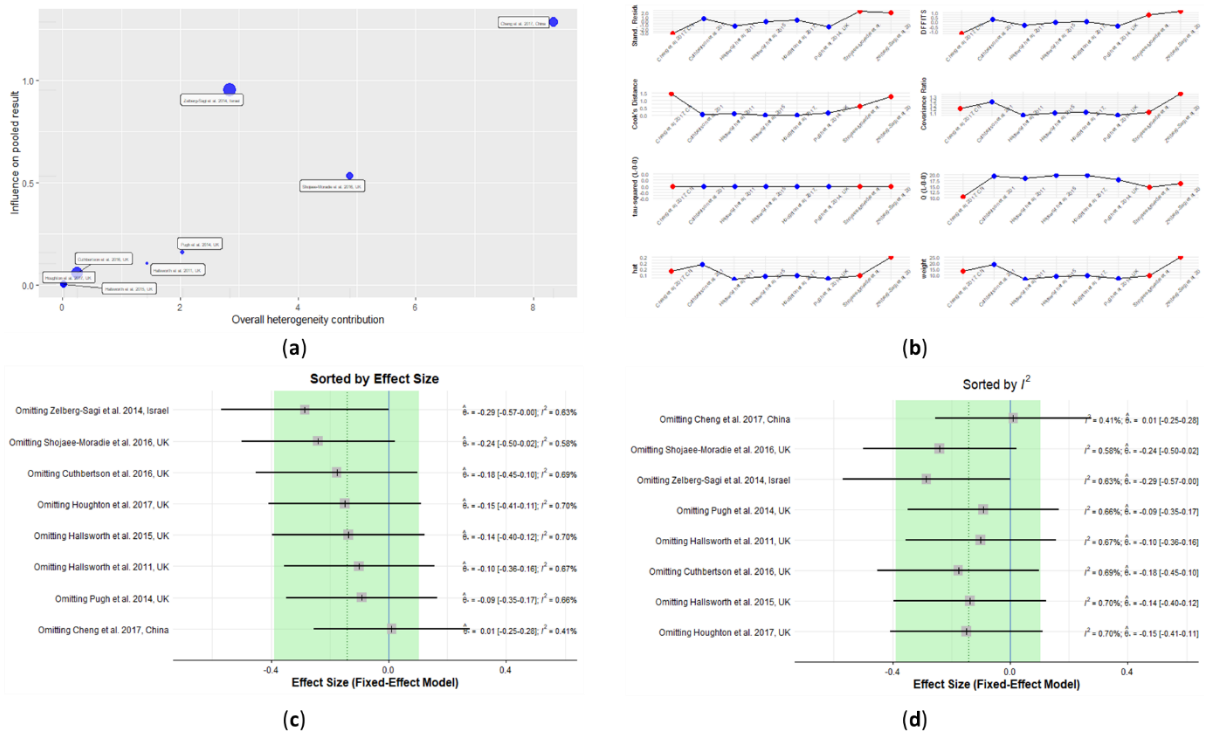

Figure S24: Influence analysis plots for fasting glucose. (**a**) Baujat plot; (**b**) Diagnostic tests for influence measures; (**c**)Leave-one-out analyses sorted by decreasing order of effect size; (**d**) Leave-one-out analyses sorted by decreasing order of heterogeneity.

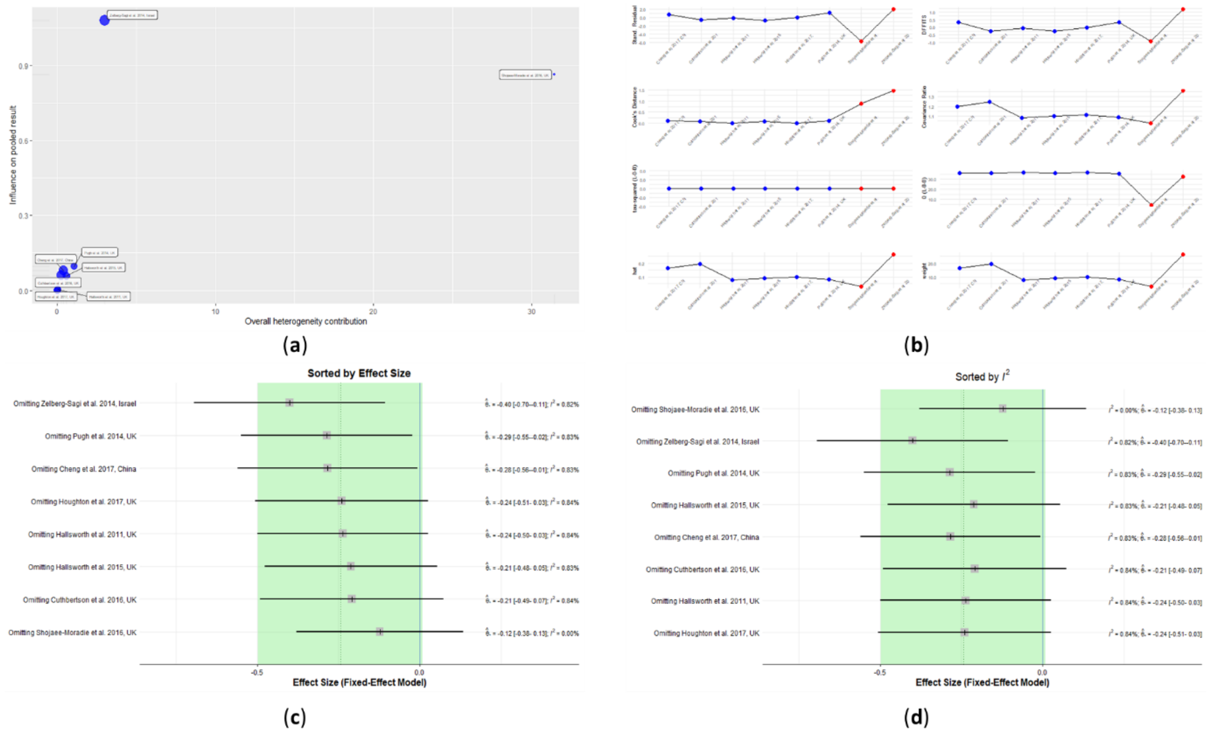

Figure S25: Influence analysis plots for fasting insulin. (**a**) Baujat plot; (**b**) Diagnostic tests for influence measures; (**c**)Leave-one-out analyses sorted by decreasing order of effect size; (**d**) Leave-one-out analyses sorted by decreasing order of heterogeneity

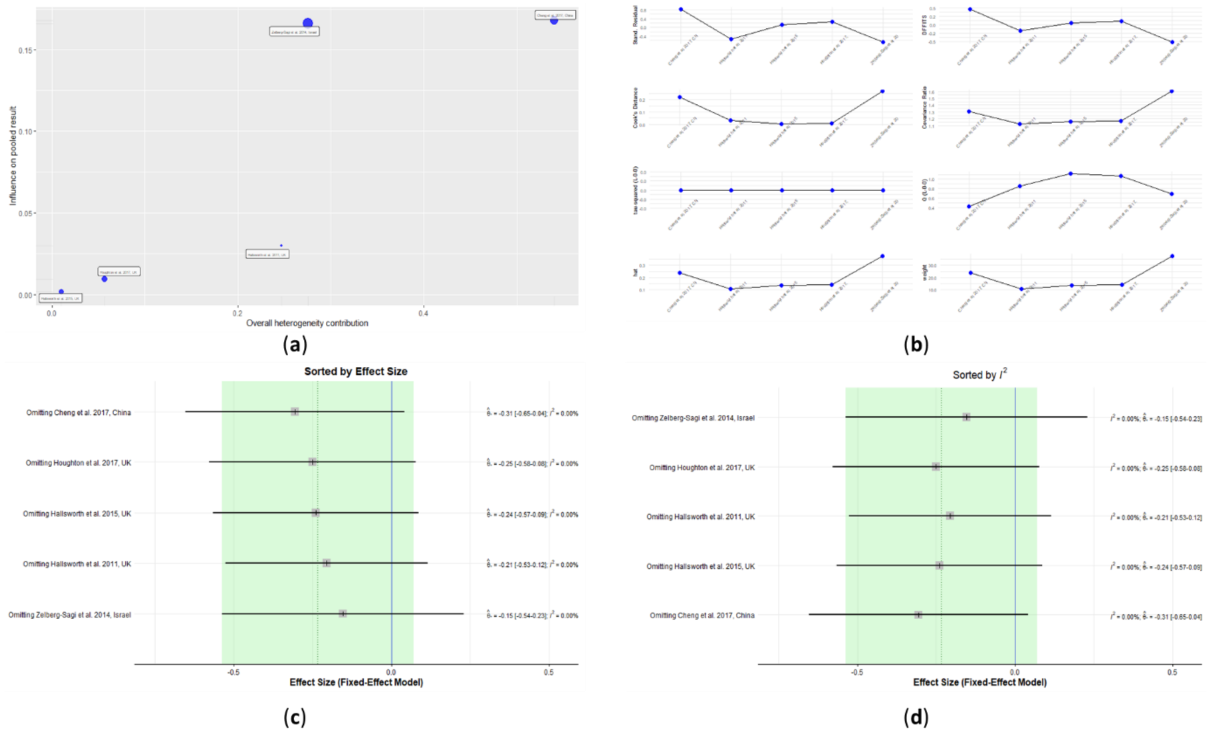

Figure S26: Influence analysis plots for glycated hemoglobin (HbA1c). (**a**) Baujat plot; (**b**) Diagnostic tests for influence measures; (**c**)Leave-one-out analyses sorted by decreasing order of effect size; (**d**) Leave-one-out analyses sorted by decreasing order of heterogeneity.

Supplementary Figure S26: Supplementary figure for forest plot

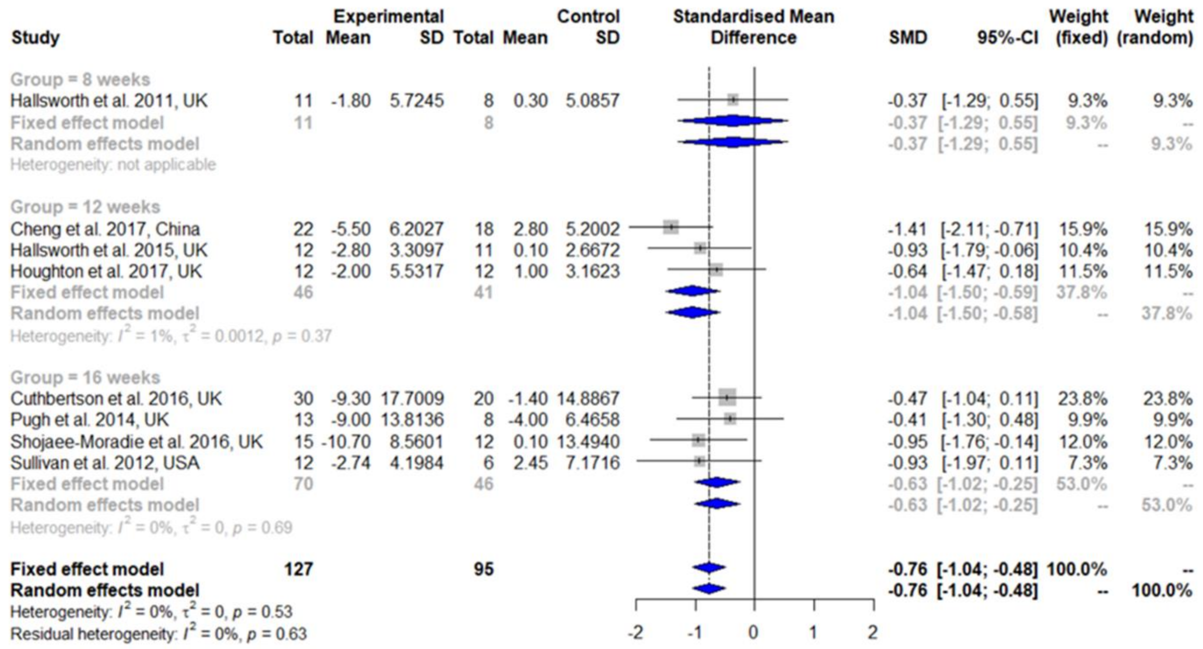

Figure S27: Forest plot of intrahepatic lipid (IHL) content with subgroup analysis based on exercise intervention duration; SD: standard deviation, SMD: standardized mean difference, CI: confidence interval
